# Exercised-Induced Coronary Spasm in Near Normal Coronary Arteries

**DOI:** 10.1155/2010/207479

**Published:** 2010-06-17

**Authors:** Damian Franzen, Thomas Benzing

**Affiliations:** ^1^Praxis für Herz und Lungenkrankheiten, Berrenratherstr. 296, 50937 Köln, Germany; ^2^Klinik IV für Innere Medizin, Universitätskliniken Köln, 50924 Köln, Germany

## Abstract

In contrast to effort-induced symptoms in obstructive coronary disease, spasm in normal coronary arteries is characterized by angina at rest. We describe a 44-year-old patient with minor coronary plaques and pure exercised-induced coronary spasm. The case questions the differential pathogenic considerations of variant of the variant as opposed to Prinzmetal's variant angina.

## 1. Introduction

As outlined by Maseri and coworkers, coronary artery spasm is a potential cause of angina pectoris in patients with and without atherosclerotic coronary heart disease [1]. Whereas spasm in obstructive coronary artery disease (termed variant angina) may be quite common, angina in (near) normal coronary arteries (called the variant of the variant angina) is rare and likely to be misdiagnosed [2]. Although the clinical picture in variant of the variant angina may vary considerably, anginal symptoms occur spontaneously and typically at rest. Symptoms may be accompanied by ST-depression or occasionally by ST-segment elevation in the electrocardiogram, however exercise tests are usually negative [3]. In the following we report on a case of pure exercise induced coronary spasm in a 44-year-old man.

## 2. Case Presentation

In June 2008, a 44-year-old salesman started to suffer from chest discomfort during hiking and bicycle riding. However, bicycle stress testing was negative. He was nonsmoker and his LDL-cholesterol was normal (101 mg/dL). Because of recurrent episodes of effort-related thoracic pain alleviated by sublingual nitroglycerine and a family history of coronary heart diseases in both parents, the patient underwent cardiac catheterization in July 2008, demonstrating near normal vessels with minor and nonobstructive sclerotic lesions at the left anterior descending and the right coronary arteries. Because of mild hypertension during exercise, he was treated with ramipril 2,5 mg daily. 

**Figure 1:**
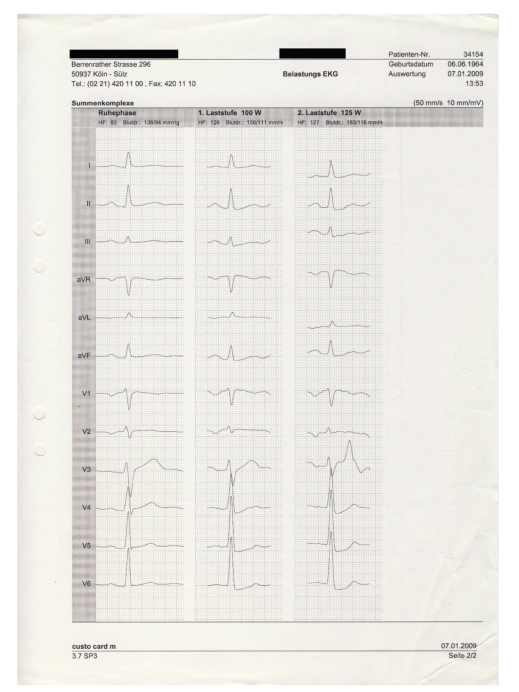
12-lead electrocardiogram demonstrating significant ST-depression in the posterior leads during exercise.

**Figure 2:**
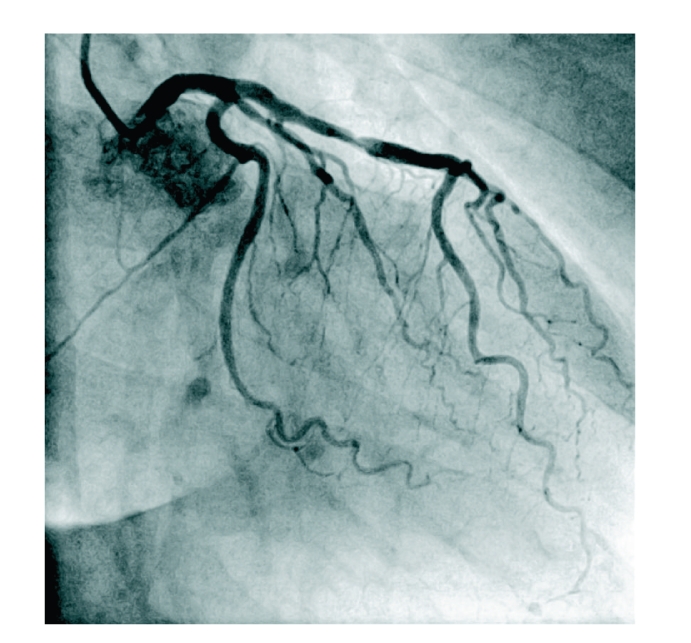
Left coronary artery (RAO 31, caudal 0,4).

**Figure 3:**
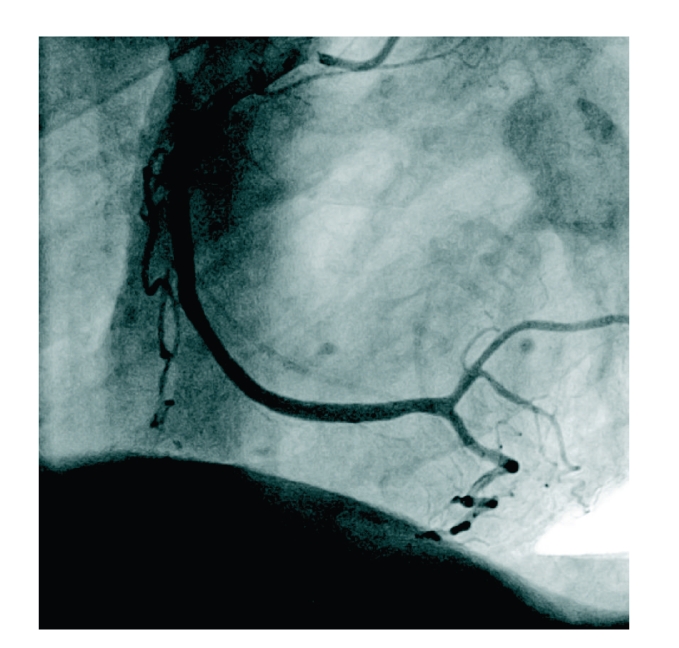
Right coronary artery (LAO 53, caudal 0,3).

**Figure 4:**
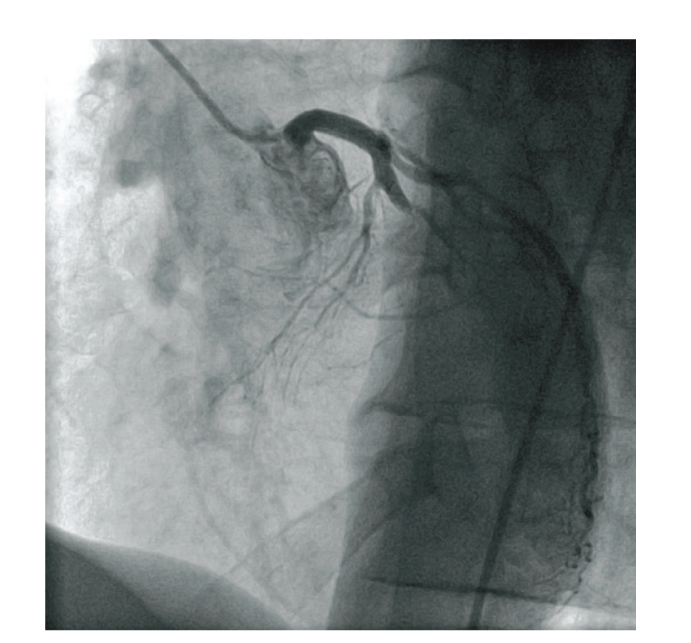
Spontaneous occlusion of the left anterior descending coronary artery (LAO 35, cranial 18) accompanied by ST-segment elevation.

**Figure 5:**
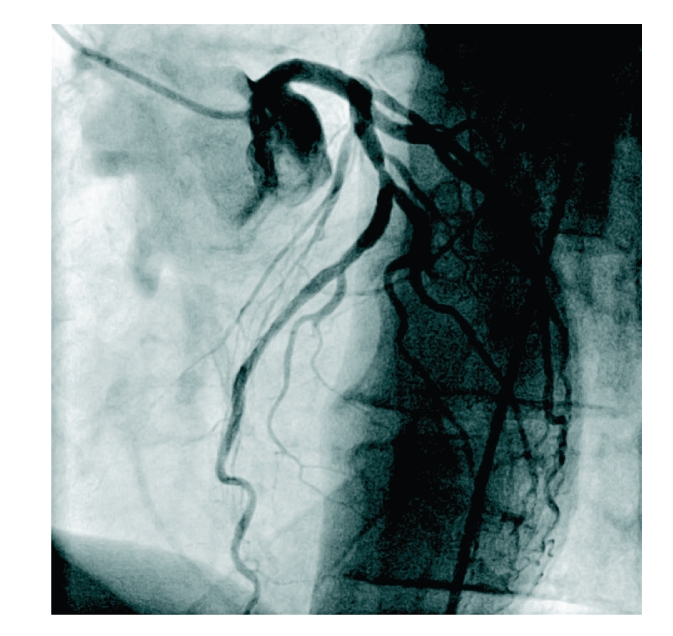
Resolution of spasm following intracoronary nitroglycerine (LAO 35, cranial 18).

As symptoms increased in intensity and frequency, another stress test in January 2009 at our institution revealed exercise induced ST-depression in leads V4–V6 accompanied by typical anginal symptoms (Figure 1). A second cardiac catheterization demonstrated a mild stenosis in the mid portion of the left anterior descending coronary artery (LAD), all other coronary arteries were without visible stenosis (Figures 2 and 3). During the procedure the patient suddenly complained of anginal symptoms accompanied by ST elevation and finally ventricular tachycardia. The LAD was found occluded beginning at the stenotic segment of the mid LAD (Figure 4). All symptoms and the spasm resolved spontaneously (Figure 5).

**Figure 6:**
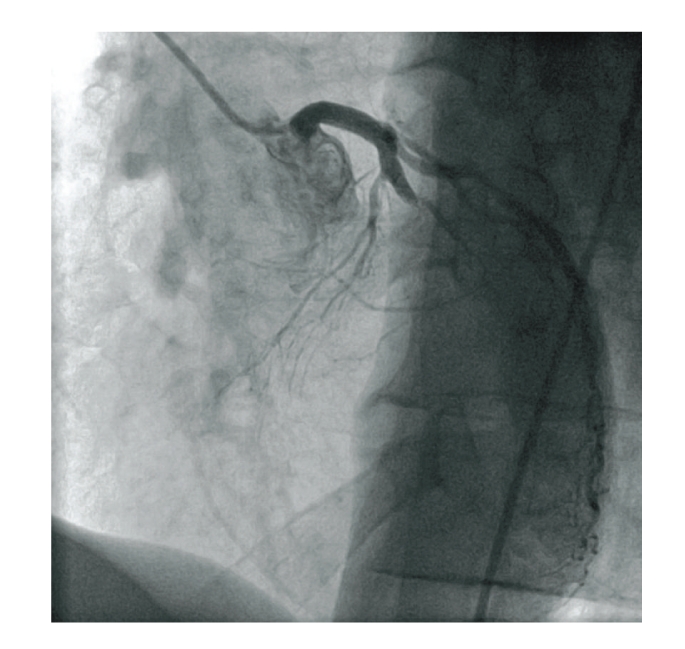
Occlusion of the left anterior descending coronary artery following intracoronary acethylcholine infusion (LAO 35, cranial 18) accompanied by ST-segment depression and T-wave inversion.

In the following, acetylcholine was infused into the left coronary artery resulting in complete occlusion of the LAD accompanied by angina and ST-depression (Figure 6) resolving directly to intracoronary nitroglycerine.

The patient was put on long-term medication with isosorbide dinitrate and amlodipine and was free of symptoms thereafter.

## 3. Discussion

Obstructive atherosclerotic lesions are the leading cause of symptomatic coronary heart disease throughout the Western civilization. As opposed to effort-induced symptoms due to stenotic coronary disease, Prinzmetal reported about a special cohort of patients with angina at rest (variant angina). He speculated on an increased vascular tone at the site of coronary plaques which had been found at postmortem studies [4]. Myocardial ischemia in normal coronary arteries has been initially documented by Cheng and coworkers [2]. Based on the clinical similarities to the variant angina of Prinzmetal, this syndrome has been termed the variant of the variant. Cheng concluded that spasm of normal coronary arteries was the most likely explanation for the observed complete relief of symptoms and coronary dilatation following nitroglycerine application. In clinical settings, spasm can be diagnosed by intracoronary provocation with ergonovine or acethylcholine [3, 5, 6] and should be separated from mechanical induced spasm due to catheter manipulation at the coronary ostia or traumatic intracoronary manipulation during cardiac catherization.

Coronary spasm in normal coronary arteries occurs mostly at rest, in rare cases at rest and during exercise [7, 8]. In the presented patient, anginal symptoms during every day life were provoked by exercise only and accompanied by ST-depression. Although symptoms indicated severe underlying organic vascular obstruction, coronary angiography demonstrated coronary spasm of the left anterior descending coronary artery. It has been speculated that coronary spasm may be related to an abnormal vasoconstrictor stimulus or to a local arterial hypersensitivity to physiologic stimuli [9–11]. 

It seems mandatory for the spastic vascular response to a generalized stimulus to be based on an pathologic substrate within a particular segment of the arterial wall. Although numerous articles on spasm in symptomatic patients without angiographic detectable coronary disease have been published, imaging with newer technologies such as intracoronary ultrasound and coronary tomography question whether spasm is an entity on its own (variant of the variant) or always related to the presence of atherosclerosis (Prinzmetal's variant angina) [12]. The presented case of effort-induced spasm in a mildly diseased coronary artery system seems to support this suggestion.

## References

[1] Maseri A, Severi S, Denes M (1978). “Variant” angina: one aspect of a continuous spectrum of vasospastic myocardial ischemia. Pathogenetic mechanisms, estimated incidence and clinical and coronary arteriographic findings in 138 patients. *The American Journal of Cardiology*.

[2] Cheng TO, Bashour T, Kelser GA, Weiss L, Bacos J (1973). Variant angina of Prinzmetal with normal coronary arteriograms. A variant of the variant. *Circulation*.

[3] Cheng TO (2007). Ergonovine test for coronary artery spasm. *International Journal of Cardiology*.

[4] Prinzmetal M, Kennamer R, Merliss R, Wada T, Bor N (1959). Angina pectoris I. A variant form of angina pectoris. Preliminary report. *The American Journal of Medicine*.

[5] Coma-Canella I, Castano S, Macías A, Calabuig J, Artaiz M (2006). Ergonovine test in angina with normal coronary arteries. Is it worth doing it?. *International Journal of Cardiology*.

[6] Ong P, Athanasiadis A, Hill S, Vogelsberg H, Voehringer M, Sechtem U (2008). Coronary artery spasm as a frequent cause of acute coronary syndrome. The CASPAR (Coronary Artery Spasm in Patients with Acute Coronary Syndrome) study. *Journal of the American College of Cardiology*.

[7] Specchia G, de Servi S, Falcone C (1979). Coronary arterial spasm as a cause of exercise-induced ST-segment elevation in patients with variant angina. *Circulation*.

[8] Freeman SB, Richmond DR, Kelly DT (1983). Clinical studies of patients with coronary spasm. *American Journal of Cardiology*.

[9] Yasue H, Touyama M, Kato H, Tanaka S, Akiyama F (1976). Prinzmetal’s variant form of angina as a manifestation of alpha adrenergic receptor mediated coronary artery spasm: documentation by coronary arteriography. *American Heart Journal*.

[10] Yasue H, Nagao M, Omote S, Takizawa A, Miwa K, Tanaka S (1978). Coronary arterial spasm and Prinzmetal’s variant form of angina induced by hyperventilation and tris-buffer infusion. *Circulation*.

[11] Bashour TT (1991). Vasotonic myocardial ischemia. *American Heart Journal*.

[12] Hoon YJ, Jeong MH, Choi YH Plaque components at coronary sites with focal spasm in patients with variant angina: Virtual histology-intravascular ultrasound analysis.

